# A CRISPR/Cas9-generated mutation in the zebrafish orthologue of *PPP2R3B* causes idiopathic scoliosis

**DOI:** 10.1038/s41598-023-33589-y

**Published:** 2023-04-26

**Authors:** Marian Seda, Berta Crespo, Michelangelo Corcelli, Daniel P. Osborn, Dagan Jenkins

**Affiliations:** 1 Genetics and Genomic Medicine Programme, UCL Institute of Child Health, London, WC1N 1EH UK; 2 Developmental Biology and Cancer Programme, UCL Institute of Child Health, London, WC1N 1EH UK; 3 Genetics Sections, Molecular and Clinical Sciences Institute, St George’s University of London, Cranmer Terrace, London, SW17 0RE UK

**Keywords:** Bone development, Cartilage development, Disease model, Experimental organisms, Neurogenesis, Organogenesis, Developmental biology, Genetics, Diseases, Molecular medicine

## Abstract

Idiopathic scoliosis (IS) is the deformation and/or abnormal curvature of the spine that develops progressively after birth. It is a very common condition, affecting approximately 4% of the general population, yet the genetic and mechanistic causes of IS are poorly understood. Here, we focus on *PPP2R3B*, which encodes a protein phosphatase 2A regulatory subunit. We found that *PPP2R3B* is expressed at sites of chondrogenesis within human foetuses, including the vertebrae. We also demonstrated prominent expression in myotome and muscle fibres in human foetuses, and zebrafish embryos and adolescents. As there is no rodent orthologue of *PPP2R3B*, we used CRIPSR/Cas9-mediated gene-editing to generate a series of frameshift mutations in zebrafish *ppp2r3b*. Adolescent zebrafish that were homozygous for this mutation exhibited a fully penetrant kyphoscoliosis phenotype which became progressively worse over time, mirroring IS in humans. These defects were associated with reduced mineralisation of vertebrae, resembling osteoporosis. Electron microscopy demonstrated abnormal mitochondria adjacent to muscle fibres. In summary, we report a novel zebrafish model of IS and reduced bone mineral density. In future, it will be necessary to delineate the aetiology of these defects in relation to bone, muscle, neuronal and ependymal cilia function.

## Introduction

Scoliosis is the lateral deformation and curvature of the spine which affects approximately 4% of the general population^1^. It has a complex aetiology with many potential cellular and tissue-level causes, and broadly three forms of scoliosis can be defined. Congenital scoliosis is present at birth and may result from a primary defect of the bone that constitutes the vertebrae, and indeed some mouse models of scoliosis exhibit alterations of osteoblasts and chondrogenesis^2^. Neuromuscular scoliosis is associated with neural or muscular abnormalities such as spina bifida or muscular dystrophy, which emphasises the importance of these tissues in maintaining spinal integrity. In these cases, scoliosis may arise secondarily to defects in proprioception^3^, which refers to the body’s sense of position and reaction to external stimuli. This is driven by connections between the interneurons that relay signals such as pain to motor neurons within the spinal cord, which in turn control muscle activity. Idiopathic scoliosis (IS) is not present at birth and generally develops progressively in children over the first decade of life without an overt anatomical cause. It may involve defects in the intervertebral discs and matrisome, ependymal cilia and cerebrospinal fluid flow, neuroinflammation etc.^4^. It is therefore important to define the primary cellular origins of these different forms of scoliosis.

*PPP2R3B* encodes the PR70 protein, a regulatory subunit of the heterotrimeric protein phosphatase 2A holoenzyme. Relatively little is known about PR70 function, although it was identified as interacting with the origin of replication complex component, CDC6, and has also been linked to regulation of autophagy^5–7^. PR70 has also been proposed to act as a tumour suppressor by regulating firing of DNA replication origins^8^. In this study we have used CRISPR/Cas9-mediated gene-editing to create a frameshift mutation in the zebrafish *ppp2r3b* gene. Homozygous mutant larvae appeared normal, while adolescents developed progressive kyphoscoliosis reminiscent of IS. *PPP2R3B* showed prominent expression in axial muscle in human foetuses, zebrafish embryos and adolescents. The mutant kyphoscoliosis phenotype is associated with reduced vertebral bone mineralisation and a muscular dystrophy-like phenotype. These data identify *ppp2r3b* as a new molecular target in the pathogenesis of IS in vertebrates.

## Results

### Expression of *PPP2R3B/ppp2r3b* in human foetuses and zebrafish

**Figure 1:**
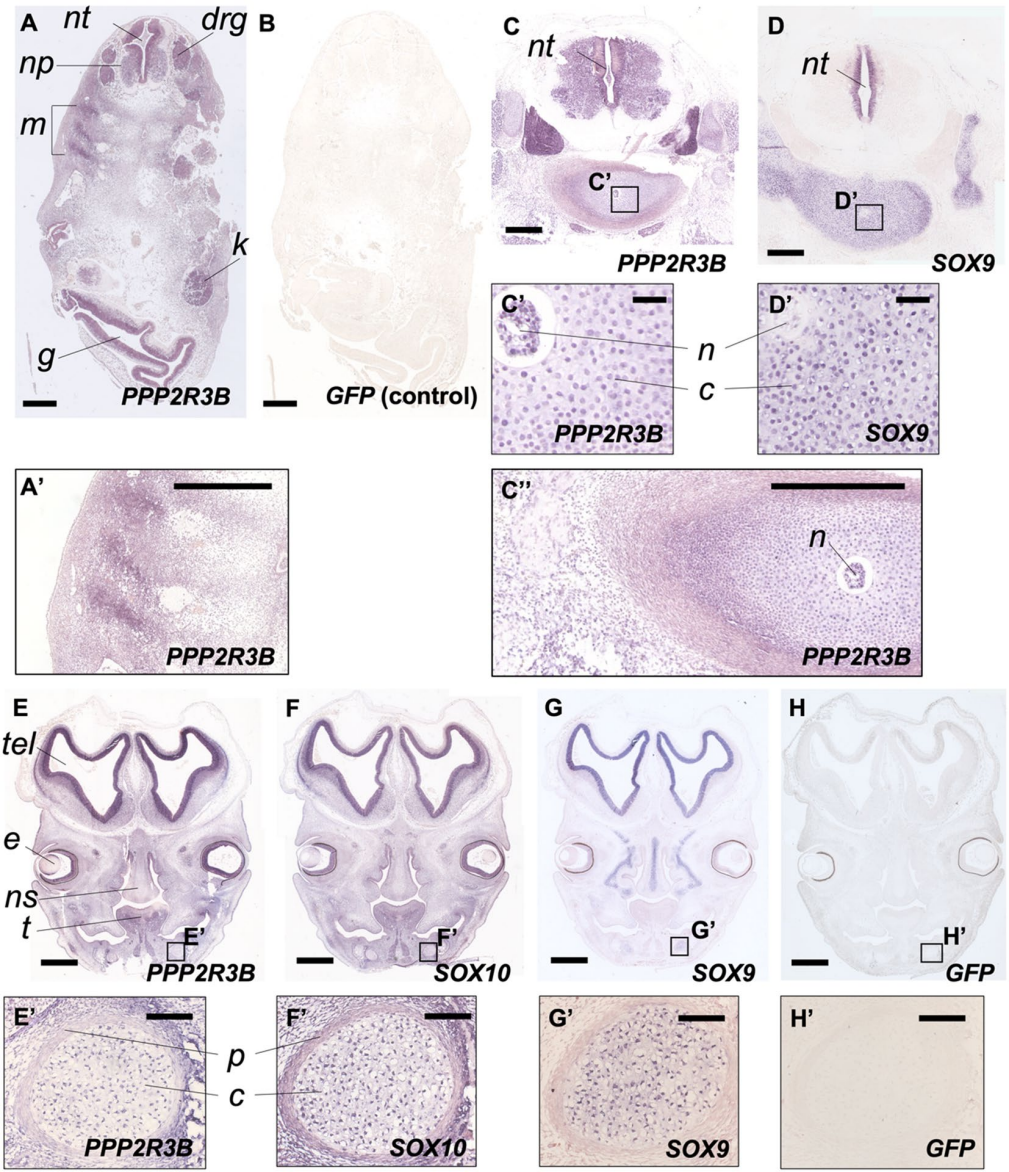
*PPP2R3B* is expressed at sites of chondrogenesis in normal human foetuses. Expression of *PPP2R3B, SOX9* and *SOX10* in normal human foetuses at Carnegie stages (CS) 17 (**A**, **B**) 23 (**C**, **D**) and 22 (**E–H**). (**A**, **B**) In situ hybridisation at low power showing expression of *PPP2R3B* within interneuron/motor neuron precursors (*np*), dorsal root ganglia (*drg*) and myotome (*m*) but no signal generated using a GFP negative control. (**C**, **D**) Expression of *PPP2R3B* in vertebral bodies. Insets show regions magnified in **C’** and **D’**. Note expression in chondrocytes (*c*). *PPP2R3B* is also expressed in the notochord (*n*) whereas *SOX9* is not. (**E–H**) Expression of *PPP2R3B* within Meckel’s cartilage (insets magnified in **E’–H’**). Note expression co-localises with *SOX10* and *SOX9* within chondrocytes (*c*) but expression is absent from perichondrium (*p*). *nt, neural tube; g, gut; k, kidney; tel, telencephalon; e, eye; ns, nasal septum; t, tongue.* Scale bars are 500 µm in (**A**–**D**, **A**’, **C**’); 60 µm in (**C**’, **D**’); 1 mm (**E**–**H**); 10 µm in (**E**’–**H**’).

To begin to investigate the requirement for PR70 in vertebrates, we looked for expression of *PPP2R3B* in human foetuses. The orientation of tissue sections used is given in Supplementary Fig. 1. Using in situ hybridisation, we noted some locations of *PPP2R3B* expression that are potentially relevant to scoliosis. This included the neural tube, dorsal root ganglia and myotome (Fig. 1A). As a control, we used a GFP antisense probe with the same length and GC content as the *PPP2R3B* probe, which gave no signal (Fig. 1B). Expression was also noted within the vertebrae as well as Meckel’s cartilage (Fig. 1C–H). *PPP2R3B* transcripts were detected within cartilage condensations suggestive of a role in chondrogenesis, although it was not expressed within the perichondrium where osteoblast precursors reside prior to their migration into the cartilage matrix (Fig. 1C’, E’). In both of these locations, *PPP2R3B* expression was similar to that of *SOX9* on adjacent sections (Fig. 1D’, G’, H’). Within Meckel’s cartilage, chondrocytes within cartilage condensations also expressed *SOX10*, as did the perichondrium (Fig. 1F’). *SOX10* is a marker of neural crest stem cells confirming the contribution of this lineage to skeletal elements within the jaw. We note that *PPP2R3B* expression was quite broad, albeit with accentuation of signal in certain locations, including myotome and vertebral chondrocytes, as shown by intermediate power images (Fig. 1A’, C’’).

**Figure 2:**
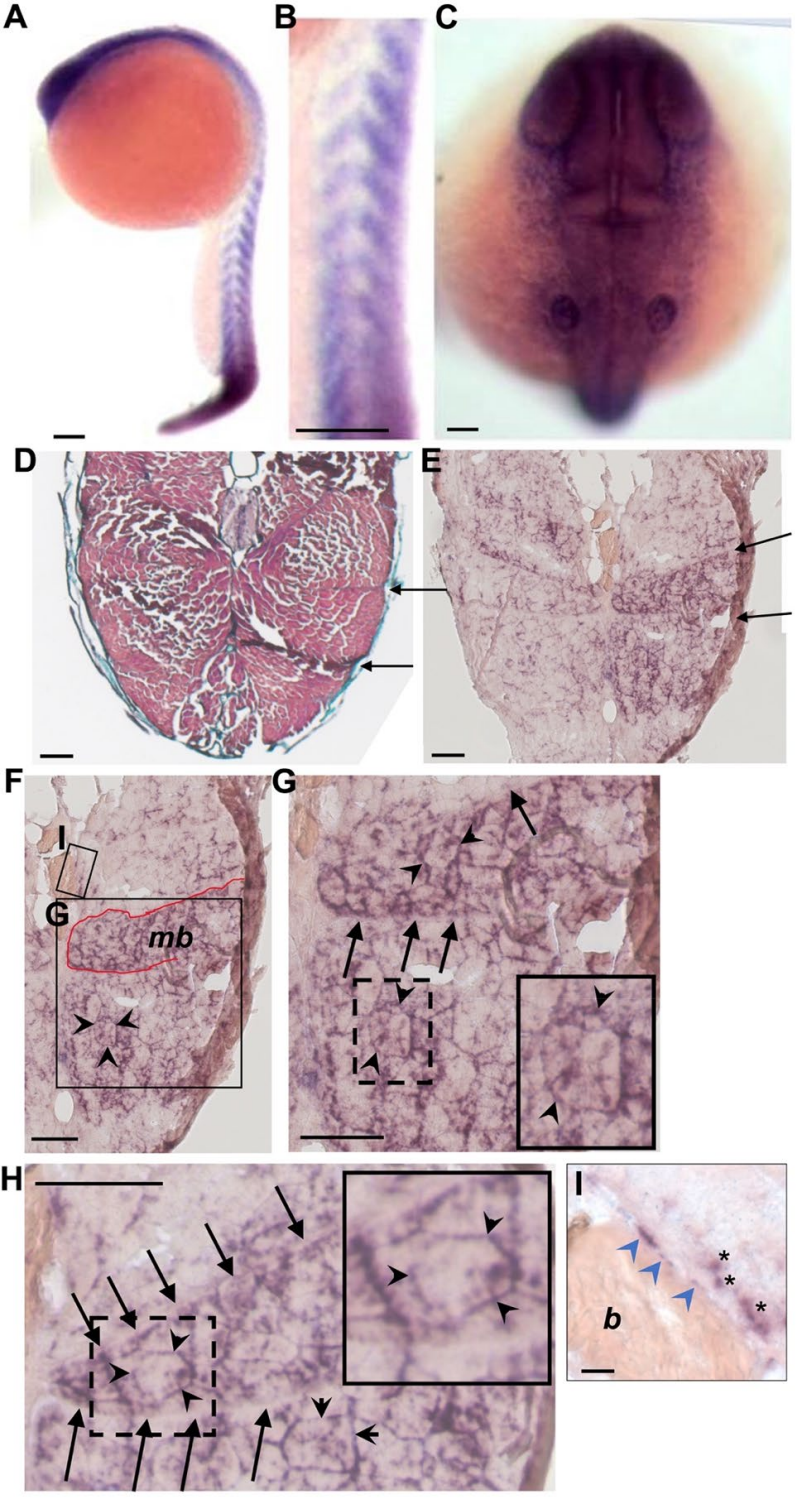
Expression of *ppp2r3b* in zebrafish. (**A–C**) In situ hybridisation showing expression of *ppp2r3b* at 24 hpf. High-power view showing expression in somites (**B**) and rostally (**C**). (**D**) Transverse section of 36 dpf zebrafish stained with Mallory’s trichrome. Arrows indicate examples of myosepta separating bundles of muscles fibres. (**E**) Similar section to (**D**) showing expression of *ppp2r3b* at 36 dpf. Arrows indicate myosepta that define muscle bundles outlined in red (*mb,* **F**). (**G**, **H**) Higher magnification images whereby arrows indicate myosepta, and arrowheads indicate staining around individual muscle fibres. Dashed boxes show the location of the insets which individual muscle fibres at higher magnification. (**I**) Inset from F showing mineralised bone (*b*) within vertebral centra showing expression of *ppp2r3b* in squamous chordoblast cells (arrowheads). Asterisks label individually staining cells of distinct morphology. Scale bars are 200 µm in (**A**, **B**); 40 µm in (**C**); 150 µm in (**D**–**H**); 12 µm in (**I**).

By aligning the human PR70 protein sequence to the zebrafish translated genome, we identified only a single orthologue with significant similarity, and the genomic locus encoding Ppp2r3b showed conserved synteny with their mammalian counterparts. Orthologues of neither *PPP2R3B* nor the adjacent gene, *SHOX*, are found in rodents. We therefore analysed expression of the orthologous zebrafish *ppp2r3b* gene by in situ hybridisation (Fig. 2A, B). At 24 h post-fertilisation (hpf) we noted repeated chevron-shaped patterns of expression along the trunk of the embryo representing the somites which will go on to form axial muscle. Within the head region, we also noted rostral expression (Fig. 2C).

We also analysed expression in zebrafish at 36 dpf of age. We noted prominent expression throughout the region in which fast twitch muscle is thought to reside, adjacent to vertebral centra (Fig. 2D–H). At this stage, a number of distinct muscle bundles separated by myospeta can be seen on transverse tissue sections (Fig. 2D). ppp2r3b is seen to be expressed prominently in these (Fig. 2E), and it is notable that staining intensity may vary between bundles, although this may relate to tissue sectioning quality (Fig. 2E). Within these muscle bundles, higher power imaging shows that ppp2r3b expression appears to surround individual muscle fibres (arrowheads, Fig. 2G, H) which is consistent with nuclear staining of multinucleate muscle fibres. In contrast there was only limited expression in proximity to mineralised bone—based on their morphology and location on vertebral bone surfaces, we did identify expression in what could be squamous chordoblast (osteoblast) -like cells, as previously reported^9^, although it is noteworthy that these cells were very rare (Fig. 2I). It should be noted that to definitively identify these cells as chordoblasts it will be necessary to identify specific markers for these cells in co-localisation studies in future.

### Generation of *ppp2r3b* mutant zebrafish using CRISPR/Cas9 gene-editing

**Figure 3:**
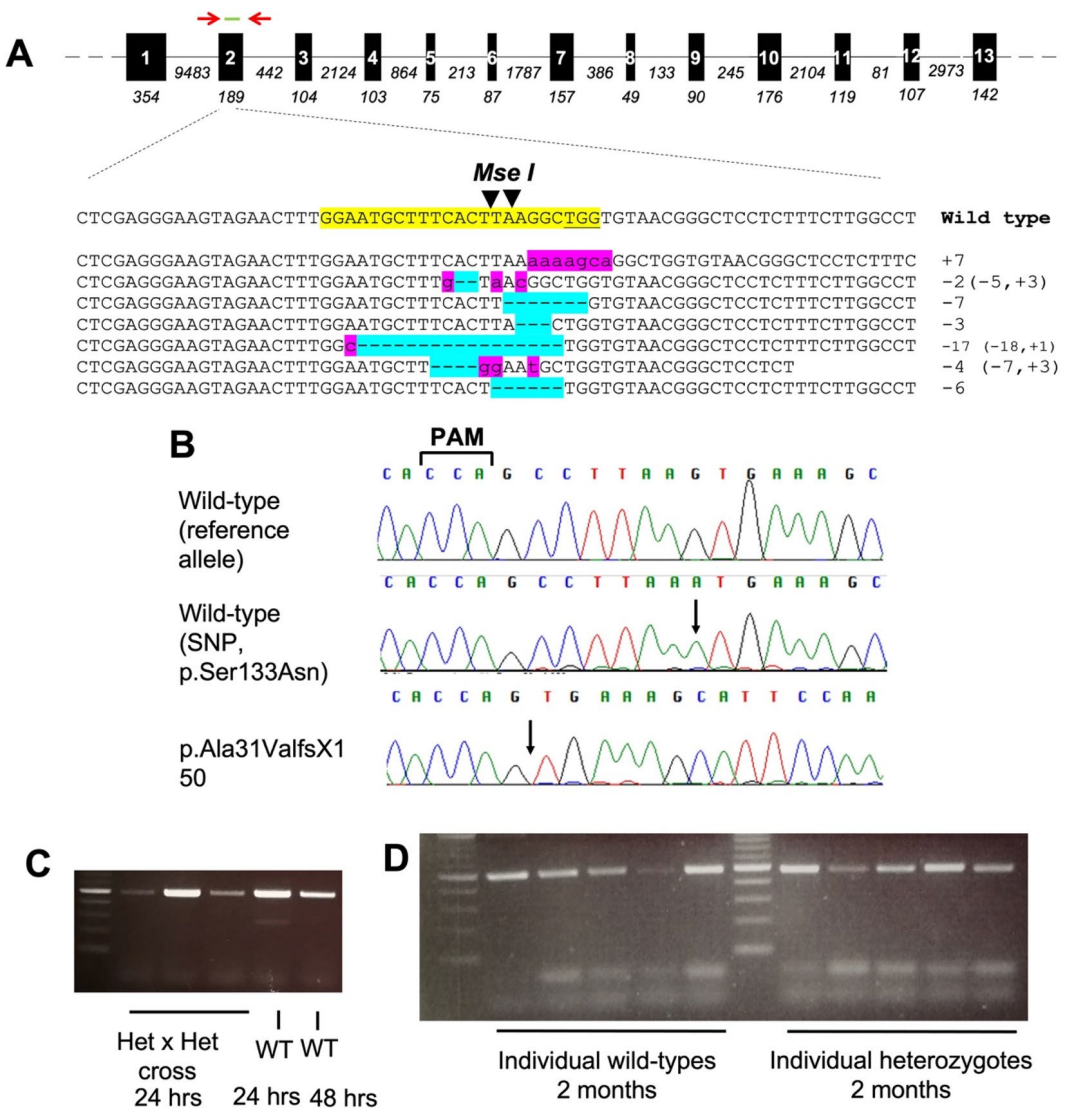
Targeting *ppp2r3b* in zebrafish using gene-editing. (**A**) *ppp2r3b* gene structure showing the location of primers used for genotyping (red arrows) and sgRNA (green) used for gene-editing. Below, examples of mutant sequence reads cloned from pooled F0 embryos. The sgRNA site is highlighted in yellow within the wild-type sequence. Inserted and deleted nucleotides are highlighted in pink and blue, respectively. (**B**) Sequence chromatograms showing the homozygous wild-type reference and alternative alleles, and the homozygous and heterozygous mutant reads. (**C**, **D**) RT-PCR showing expression of *ppp2r3b* using primers located within (**C**, **D**) exons 1–3 (band at expected size, 491 bp) or (**D**) or exons 1–7 (band at expected size, 1000 bp) at the indicated stages.

To generate a genetic model of *ppp2r3b* loss-of-function, we used CRISPR/Cas9 gene-editing to target this gene using a sgRNA located within exon 2 (Fig. 3A). This sgRNA was located on the forward strand and had no self-complementarity or predicted off-target sites according to the chopchop tool (http://chopchop.cbu.uib.no/). An *Mse I* restriction site was located within the sgRNA binding site which allowed us to monitor the efficiency with which indels were introduced at this location. Direct sequencing of a selection of cloned mutations from mosaic F0 embryos at 24 hpf was consistent with many previous reports in zebrafish, showing that CRISPR/Cas9 typically produces complex indels involving deletions of between 2 and 18 nucleotides (Fig. 3A).

**Figure 4:**
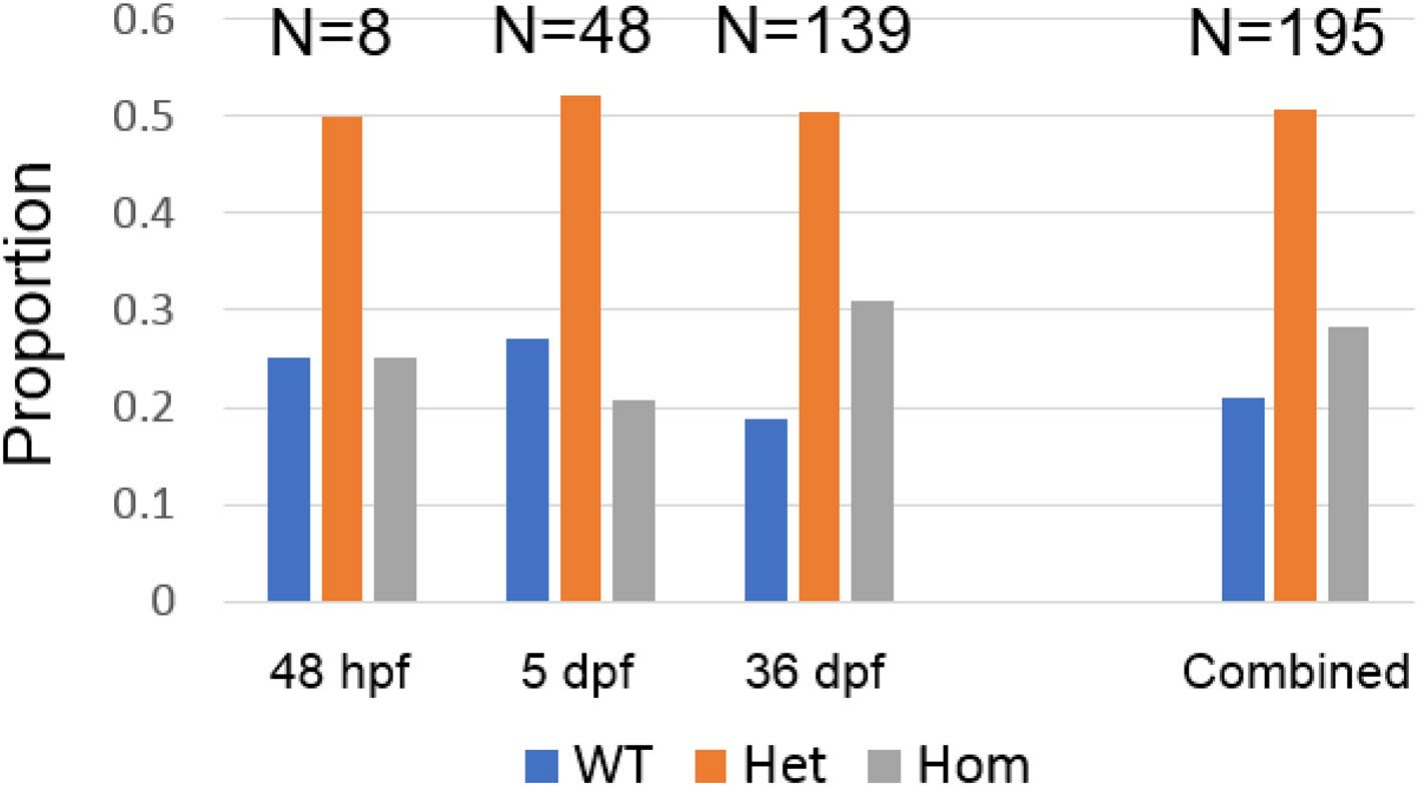
*ppp2r3b* mutants are viable at all ages. Proportions of wild-type (WT), heterozygous (Het) and homozygous (Hom) mutant (*ppp2r3b*^Ala31ValfsX150^) animals at the stated ages. Total numbers of embryos analysed are indicated.

We have now outcrossed these F0 mosaics and their progeny for more than 5 generations to achieve germline transmission and to avoid possible off-target mutations. We isolated a line of zebrafish carrying a 7 bp deletion in exon 2 of *ppp2r3b* which results in the frameshift mutation p.Ala31ValfsX150 (Fig. 3B). Homozygous mutants are hereafter referred to as *ppp2r3b*^*−/−*^. During the course of our breeding and genotyping, we noted a single nucleotide polymorphism (SNP) located within the sgRNA binding site which is not present on publicly available databases. This SNP encodes the single amino acid substitution p.Ser33Asn. In all subsequent analyses, we selected only heterozygotes whose wild-type allele encoded the reference SNP at this location in our breeding population. RT-PCR and direct sequencing of gel extracted products using primers located in exons 1–3 or 1–7 failed to identify gross alternative splicing within *ppp2r3b* transcripts generated from pooled 24 hpf embryos from a hetxhet incross or individual homozygous mutant animals at 48 dpf of age—these products were of the predicted size, as in wild-types, and qRT-PCR demonstrated that transcript levels in mutants were not different from wild-type (Fig. 3C, D, Table 1). We noted that there was no deviation from expected Mendelian ratios showing that this mutation does not affect viability (Fig. 4). We also endeavoured to generate a pool of homozygous mutant adults with which to breed maternal-zygotic mutant zebrafish. This was not possible, because homozygotes never produced any eggs, and thus we conclude that they are infertile. Notably, crosses of homozygous females with wild-type males readily produced viable fry, whereas the opposite did not.

**Table 1:** qPCR analysis of bone and muscle marker genes.

Gene	Genotype	Rel. expression	SEM	p-value
Target gene				
ppp2r3b (primer set 1)	WT	1	0.20566	0.7752
ppp2r3b (primer set 1)	ppp2r3b−/−	0.91618	0.19554	
ppp2r3b (primer set 2)	WT	1	0.20067	0.241
ppp2r3b (primer set 2)	ppp2r3b−/−	0.68974	0.14055	
Muscle marker genes				
mstnb	WT	1	0.21493	0.0512
mstnb	ppp2r3b−/−	0.42757	0.12753	
mylz2	WT	1	0.20609	0.1026
mylz2	ppp2r3b−/−	0.56843	0.11124	
smyhc1	WT	1	0.21205	0.782
smyhc1	ppp2r3b−/−	1.29499	1.00891	
stnnc	WT	1	0.34582	0.5894
stnnc	ppp2r3b−/−	1.93724	1.63087	
myhz2	WT	1	0.24932	0.8358
myhz2	ppp2r3b−/−	1.0712	0.22007	
tnni2	WT	1	0.28223	0.6054
tnni2	ppp2r3b−/−	0.8193	0.18237	
myog	WT	1	0.2499	0.6072
myog	ppp2r3b−/−	1.18719	0.2449	
tnnc1b	WT	1	0.25356	0.2269
tnnc1b	ppp2r3b−/−	0.64582	0.09455	
Bone marker genes				
ctsk	WT	1	0.17927	0.16
	ppp2r3b−/−	1.81554	0.49503	
rank l	WT	1	0.2353	0.6865
	ppp2r3b−/−	1.15204	0.27555	
col2a1a	WT	1	0.19301	0.2527
	ppp2r3b−/−	1.61816	0.4685	
mmp2	WT	1	0.2274	0.7332
	ppp2r3b−/−	0.89729	0.18151	
mmp9	WT	1	0.27651	0.2306
	ppp2r3b−/−	0.61384	0.11011	
mmp13	WT	1	0.3806	0.5372
	ppp2r3b−/−	1.36542	0.42002	
timp2a	WT	1	0.19044	0.1307
	ppp2r3b−/−	0.60948	0.13231	
spp1	WT	1	0.17135	0.6068
	ppp2r3b−/−	1.1612	0.24748	
runx2b	WT	1	0.17498	0.8269
	ppp2r3b−/−	1.05905	0.19411	
sox9b	WT	1	0.15355	0.5506
	ppp2r3b−/−	1.16335	0.21253

### *ppp2r3b* homozygous mutant zebrafish exhibit a fully penetrant scoliosis phenotype

**Figure 5:**
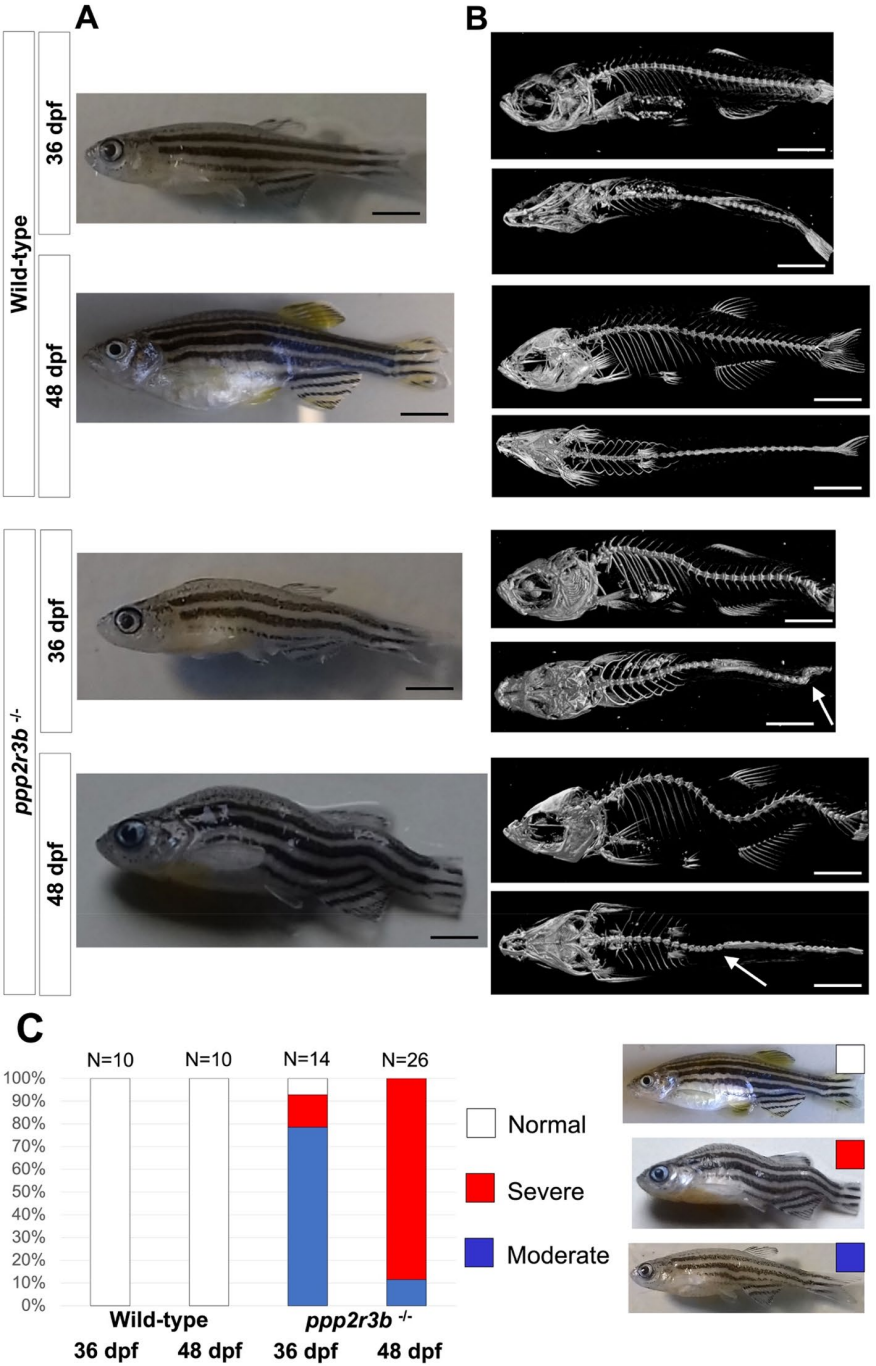
(**A**) Bright field images and (**B**) microCT scans of representative wild-type or homozygous *ppp2r3b*^−/−^ mutant zebrafish at 36 or 48 dpf. White arrows in (**B**) point to sharp lateral curvatures of the spine. (**C**) Quantification of the proportion of animals with mild, moderate or severe kyphoscoliosis. Numbers of animals are given. Scale bars are 5 mm. All animals were generation F4.

We did not identify any phenotypic abnormalities in heterozygous or homozygous mutants at larval stages. At 48 dpf, we noted that homozygotes developed severe kyphoscoliosis (Fig. 5A, B). The pattern of kyphoscoliosis was very stereotypical, characterised by two ventral curves located within the precaudal and caudal vertebrae at numbers 7–9 and 25, respectively. These ventral curves flanked a dorsal curve located at approximately caudal vertebrae number 18. There was often also a sharp lateral bend within the caudal fin, although this was not as consistent. At this age, wild-type siblings never exhibited kyphoscoliosis and the spine exhibited a very gentle ventral curvature within the precaudal region only. Scoliosis is a common phenotype in old zebrafish, presenting in excess of 18 months of age in our aquatics facility but never earlier than this. This is often associated with *Mycobacterium chelonae* infection, however, ongoing microbiological testing confirms that this species is absent from our facility.

**Figure 6:**
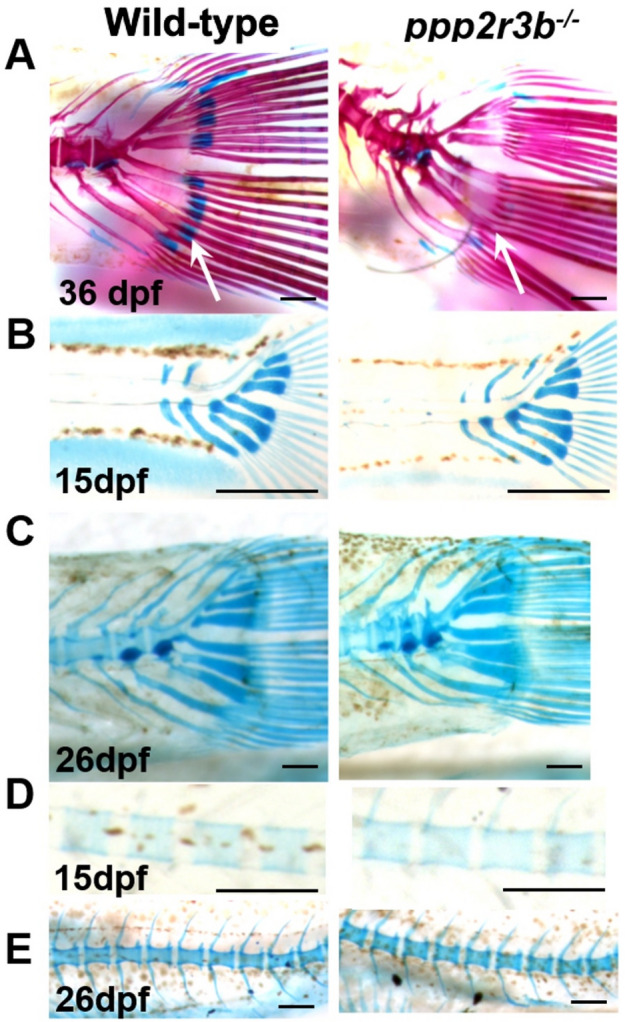
Temporal analysis of cartilage. Articular cartilage within the hypural bones is lost by 36 dpf (**A**, arrows) in *ppp2r3b*^*−/−*^ mutants but forms normally at 15 dpf (**B**) and is maintained until 26 dpf (**C**). Within the vertebral bodies, cartilage is induced and maintained normally (**D**, **E**) and is replaced by mineralised bone by 36 dpf (**A**, and data not shown) in mutants. Scales bars are (**A**) 2.5 mm, (**B**) 0.5 mm, (**C**) 2.5 mm, (**D**) 150 µm, (**E**) 5 mm.

We monitored the onset and progression of scoliosis in *ppp2r3b*^*−/−*^ mutants. Scoliosis was first seen at 36 dpf (Fig. 5)—no axial defects were observed before this as we stained animals for Alizarin red and Alcian blue as early as 15 dpf (Fig. 6). At 36 dpf, the typical presentation was moderate ventral curvature within the precaudal region, with relatively little curvature of the caudal vertebral regions. However, by 48 dpf, the final pattern consisting of two ventral curves and one dorsal curve was apparent. Quantification of the proportion of animals with moderate or severe kyphoscoliosis confirmed that this phenotype became worse with time (Fig. 5C). At 48 dpf, the kyphoscoliosis phenotype was present in all homozygous mutants, but not in any wild-type or heterozygous siblings. Therefore, *ppp2r3b*^*−/−*^ mutant zebrafish exhibit adolescent onset and progressive kyphoscoliosis, which is fully penetrant and reminiscent of human IS.

**Figure 7:**
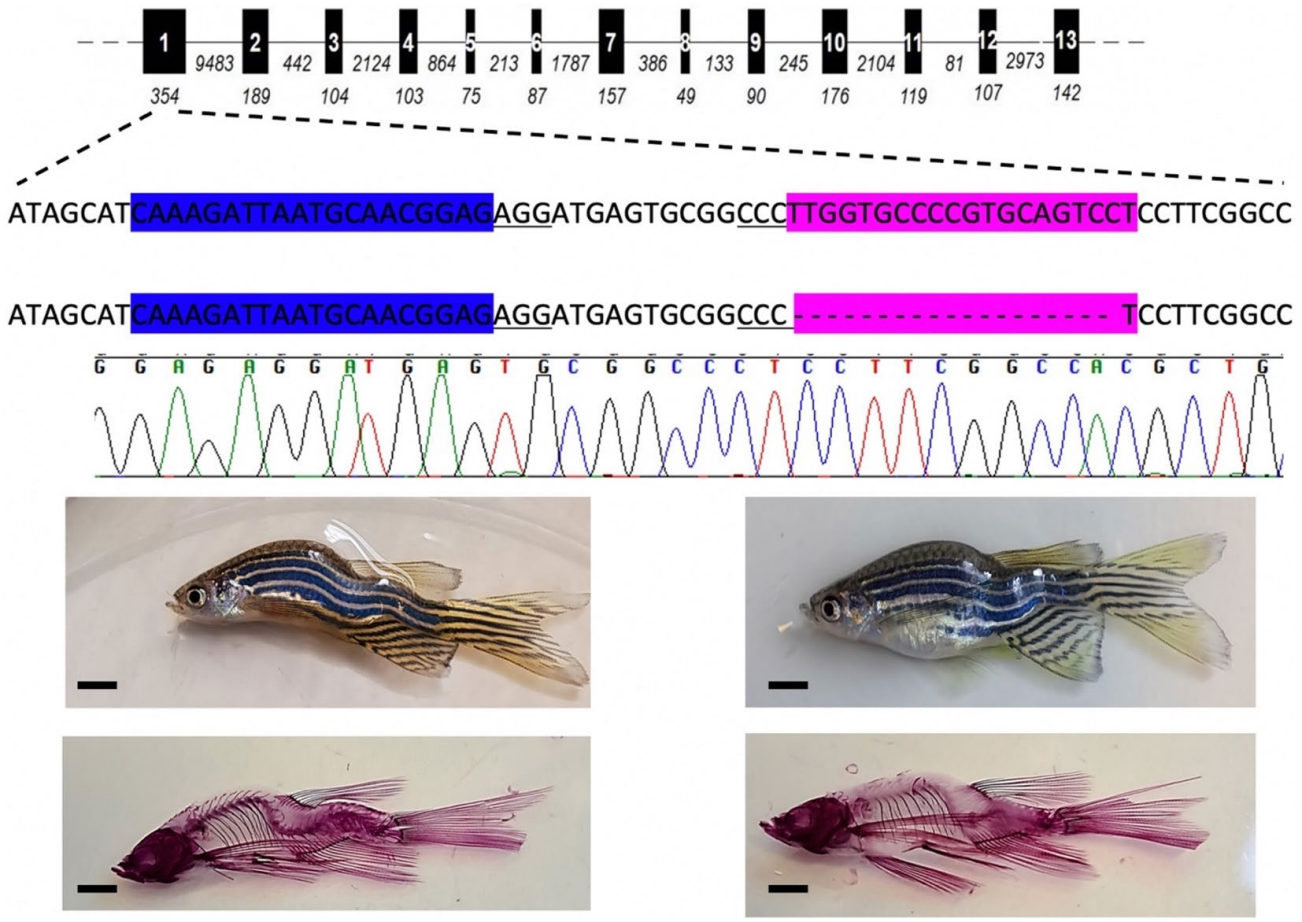
Scoliosis replicated in a mutant line encoding the mutation p.L82fsX24. Schematic of *ppp2r3b* showing the genic location of two sgRNAs used to target exon 1, highlighted in blue and pink, respectively. Deletion of 19 bp is indicated by ‘-’ symbols and the confirmatory sequence chromatogram is shown. Representative examples of two 3 month old zebrafish (above) stained for Alizarin red (below). All animals are generation F4. Scale bars are 5 mm.

To confirm that this phenotype is not the product of off-target mutations we also generated a second frameshift mutant line following microinjection of a ribonucleoprotein complex consisting of sgRNAs targeting exon 1 in complex with Cas9. This generated a 19 bp deletion, causing an out-of-frame p.L82fsX24 mutation. Notably, homozygous zebrafish for this mutation also exhibited profound scoliosis (Fig. 7).

### Kyphoscoliosis in *ppp2r3b* mutants is associated with reduced bone mineralisation of vertebrae

**Figure 8:**
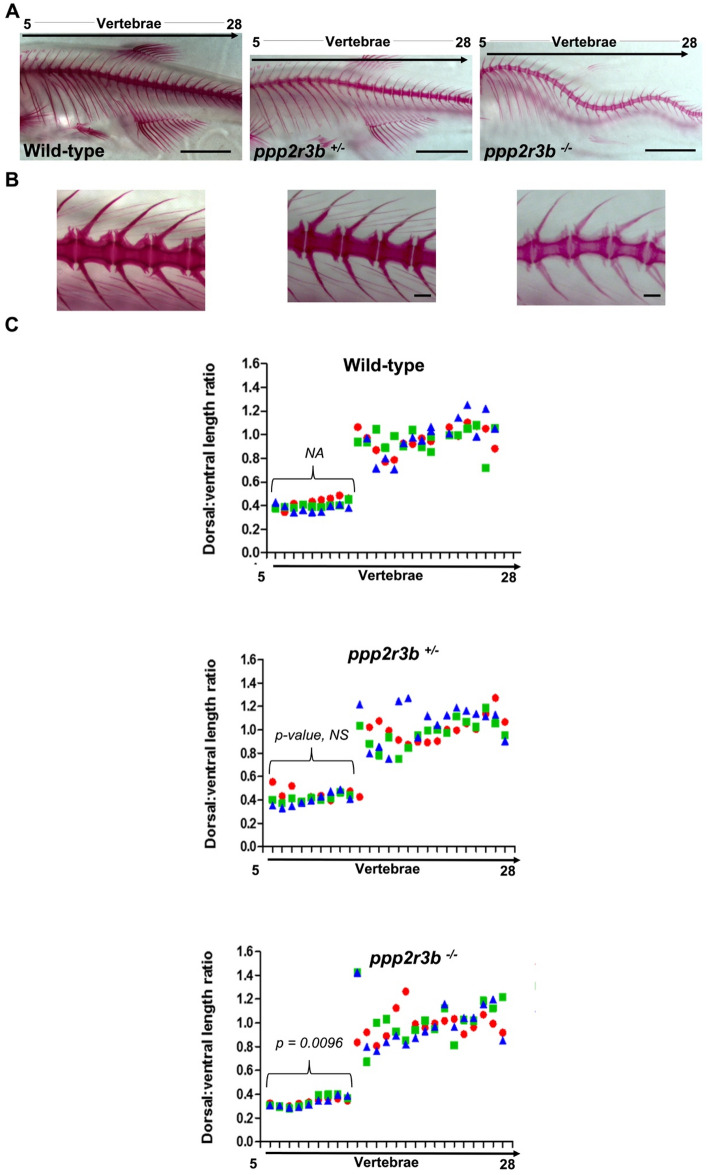
Morphometric analysis of mineralised bone. (**A**, **B**) Alizarin red staining of vertebrae 36 dpf zebrafish of the indicated genotypes. (**C**) Quantification of the neural spine:hemal spine/rib length ratios for vertebrae 5–28. Data-points for individual animals are indicated by different colours. Mean ± standard deviation of values of these ratios averaged across all ribs for each animal, indicated by the brackets, and subsequently averaged over three animals are 0.401 ± 0.024, 0.423 ± 0.029 and 0.335 ± 0.005 for wild-type, heterozygous and homozygous mutant animals, respectively (n = 3 animals). P-values compared to wild-type are indicated. This represents a statistically significant difference in homozygotes versus each of the other two genotypes (*t-test*). Tail lengths taking into account spinal curvature were 12.694, 12.872 and 12.749 mm for the representative wild-type, *ppp2r3b*+*/−* and *ppp2r3b−/−* animals shown, respectively. All animals are generation F4. Scale bars are 5 mm in (**A**); 2 mm in (**B**).

To investigate this phenotype further, we analysed bone mineralisation and cartilage formation in *ppp2r3b*^*-/-*^ mutants. Precaudal vertebrae 5–13 include a neural spine, which projects dorsally, and two ventrally located ribs, while the caudal vertebrae include neural and haemal spines which mirror one another in size. Alizarin red staining showed that the ratio between the lengths of the neural and haemal spines within the caudal region were approximately equal in length in homozygous mutants as in wild-types and heterozygotes (Fig. 8A–C). Within the precaudal region, the ribs are approximately 2.5 times longer that the neural spines (dorsal:ventral ratio of 0.4), however, we found that the ribs were relatively shorter in *ppp2r3b* homozygotes as compared to wild-types or heterozygotes (Fig. 8C), suggesting a defect in patterning and/or ossification. We also noted a marked reduction in Alizarin red staining intensity throughout the vertebral body and spines/arches, which was uniform across all vertebrae in caudal and precaudal regions (Fig. 8B).

**Figure 9:**
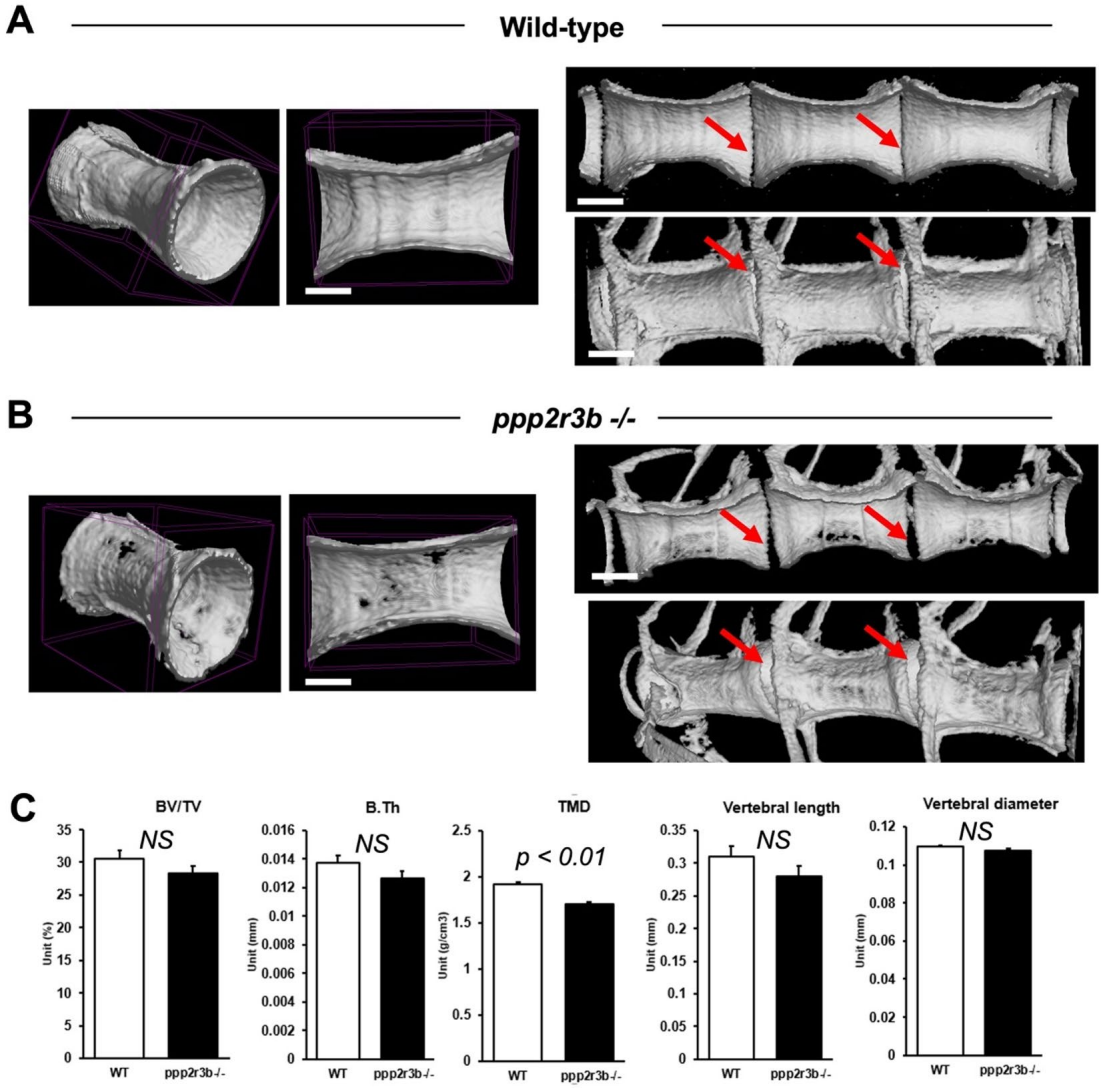
Reduced mineral density of vertebral cortical bone in *ppp2r3b*^*−/−*^ mutants. (**A**, **B**) Representative 3D and cross-sectional images from microCT scans of vertebrae in wild-type and *ppp2r3b*^*−/−*^ mutant zebrafish at 36 dpf. Note holes are apparent in the mutant vertebrae. Red arrows indicate intervertebral discs. (**C**) Quantification of a selection of cortical bone parameters measured in wild-type and *ppp2r3b*^*−/−*^ mutant vertebrae (*n* = *3*). BV/TV, bone volume/tissue volume; B.Th, bone thickness; TMD, tissue mineral density. *p-values* are given (*t-test*). NS, not significant. Average tail length (TL) for both mutant and wild-type animals was 12.6 mm with no difference between the two groups (*p-*value = 0.15, *t-test*). TLs of the two representative animals shown in (**A**) and (**B**) were 12.912 and 12.907 mm, respectively. Animals are generation F4. Scale bars are 5 mm in (**A**, **B**) left panel; 3.75 mm (**A**, **B**) right panels.

**Figure 10:**
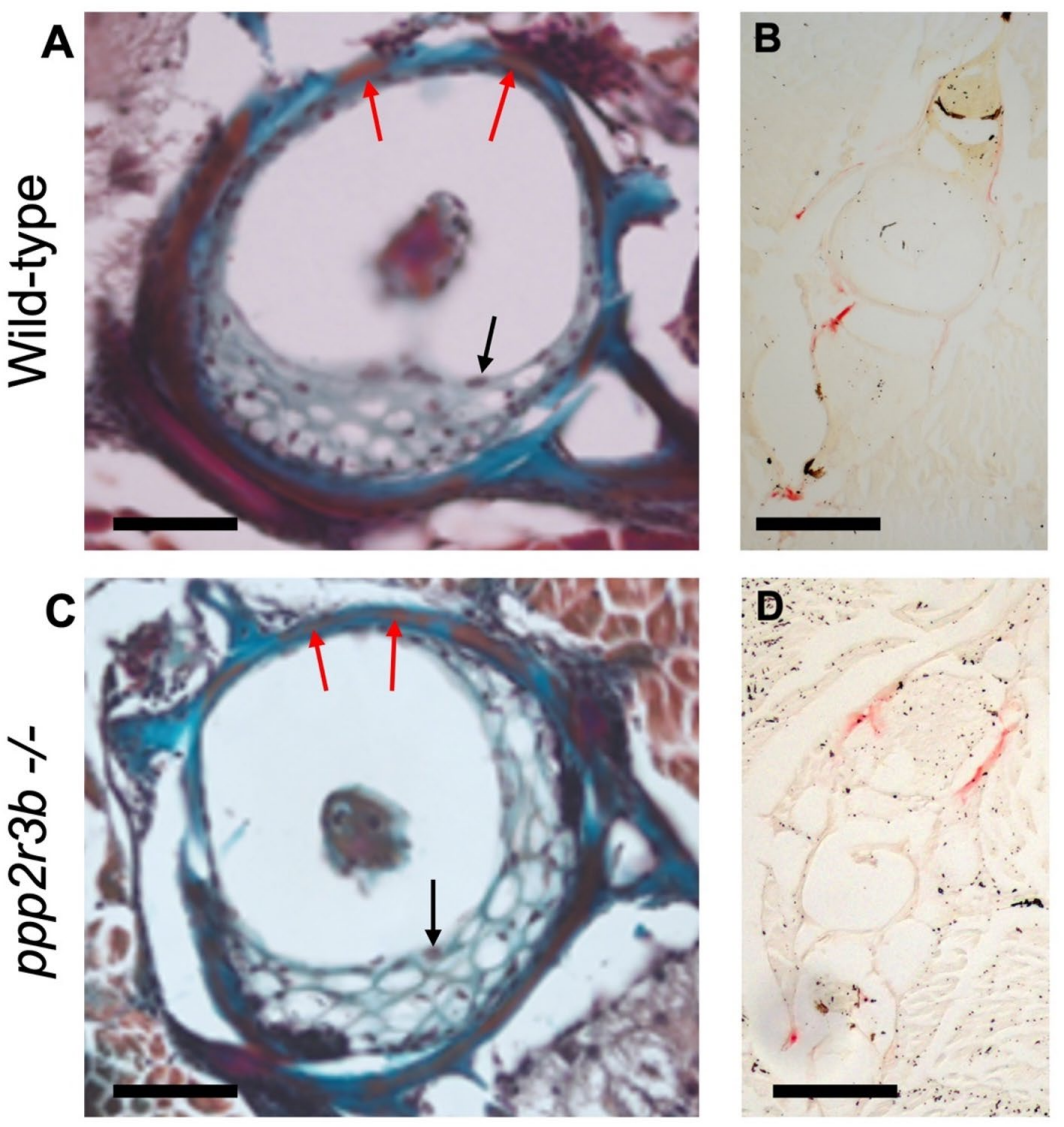
No obvious changes in osteoclast or osteoblast staining in vertebrae. (**A**, **C**) Mallory’s trichrome staining of vertebra centra. Black arrows show examples of vacuolated chordoblasts (osteoblasts) within the vertebra centrum while red arrows show squamous chordoblasts within the notochord sheath. (**B**, **D**) tartrate-resistant acid phosphatase (TRAP) staining in transverse sections from wild-type and *ppp2r3b−/−* mutant zebrafish at 36 dpf. Red signal indicates TRAP staining in neural and haemal arches, but no staining was detected within the vertebrae centra. Scale bars are 50 µm in (**A**, **C**); 14 µm in (**B**, **D**).

To investigate bone formation in more detail, we performed microCT scanning to compare skeletal tissue parameters of vertebrae at 36 dpf which represents the onset of scoliosis. This Alizarin red staining suggested that the gross structure of all vertebrae was normal without the characteristic wedging of vertebrae that has been reported previously^4^, even at the sites of curvature (Fig. 6A, B). Indeed, whereas 3D renders and longitudinal sections through contiguous vertebrae showed that adjacent vertebrae were closely apposed in wild-types, with a uniform and narrow intervertebral space, the intervertebral spaces were wedge-shaped in mutants, corresponding with the direction of curvature (Fig. 9A, B). Remarkably, we also found that multiple holes were apparent throughout the mutant vertebrae (Fig. 9A, B). Measurement of tissue mineral density (TMD), which is specific to cortical bone and is appropriate for analysis of non-trabeculated vertebral bone, was significantly reduced (Fig. 9C). However, the overall dimensions of the vertebrae, including length and diameter, were not affected suggesting a specific effect on bone mineralisation rather than morphogenesis. qRT-PCR of RNA extracted from the trunk at 36 dpf did not reveal any changes in the expression of a panel of key bone cell markers (Table 1). Histological analyses of osteoclasts and osteoblasts did not reveal differences between wild-type and mutant (Fig. 10)—Mallory’s trichrome staining showed similar numbers of vacuolated chordoblasts (osteoblasts) and squamous chordoblasts within the notochord centra and sheath, respectively. Tartrate-resistant acid phosphatase (TRAP) staining labels osteoclasts and revealed similar staining in neural and haemal arches in both wild-type and mutant, although no staining was detected within the vertebrae centra which is where the reduced TMD was observed previously. These data are subject to technical limitations relating to the proportionally small number of bone cells in whole zebrafish tissues at this stage and the non-quantitative nature of histological methods and are therefore not conclusively negative. In future, it will be necessary to analyse these cell types in more detail.

### Abnormal mitochondria associated with axial muscle in *ppp2r3b* mutants

**Figure 11:**
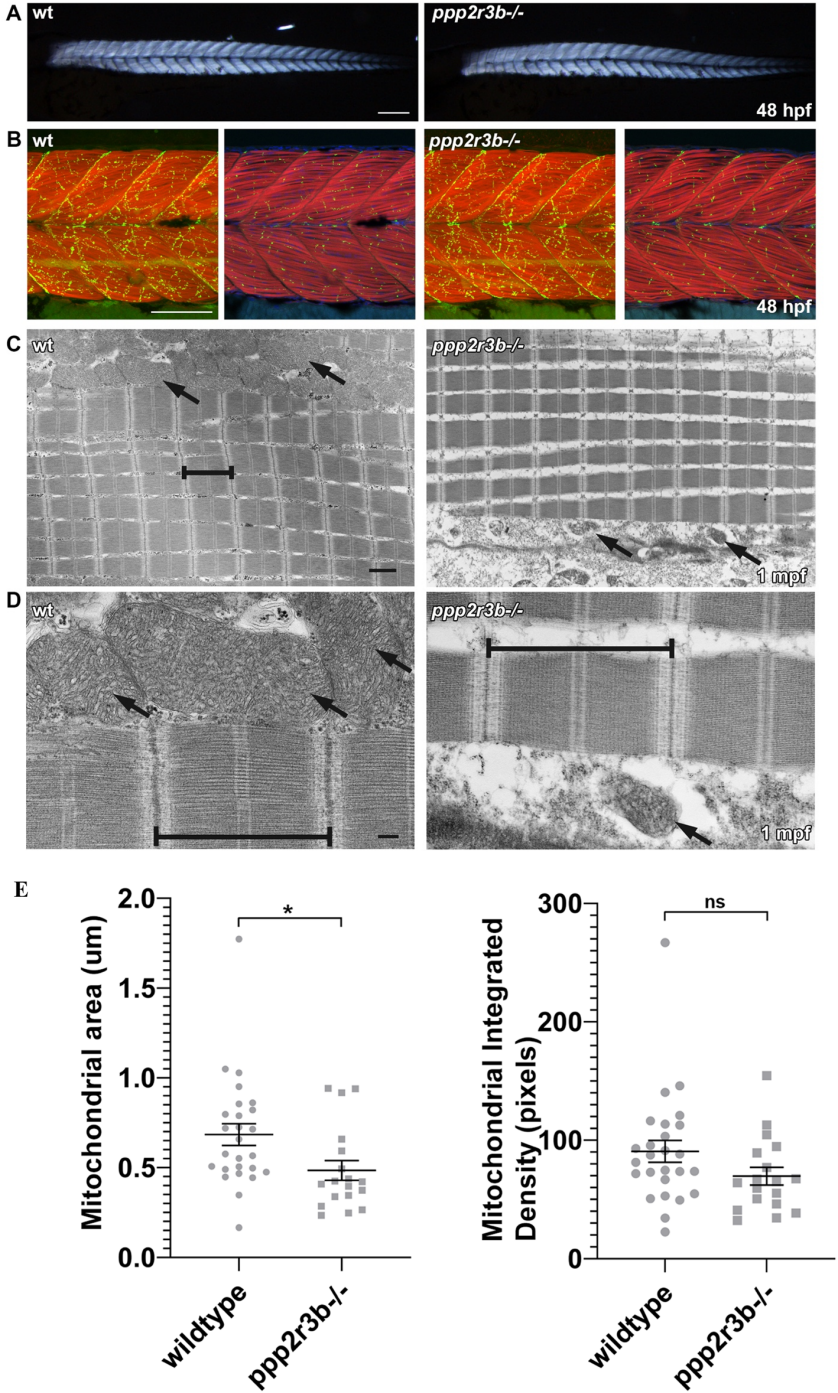
*ppp2r3b* mutants display abnormal mitochondria formation during adult stages of development, whilst muscle formation remains intact. (**A**) Skeletal muscle birefringence at 48hpf shows comparable muscle integrity between *ppp2r3b* mutants and wildtype siblings. Scale bar 200 µm. (**B**) Muscle fibres and motor neuron synapses appear normal in mutants compared to wildtype siblings at 48hpf, analysed by staining for F-Actin (Red) and Acetylcholine receptors (AChR, green) respectively. Left panels show a z-stack projection of F-Actin with AChR, right panels show a single focal plane of F-Actin/AChR/DAPI. Scale bar 100 µm. (**C**) Transmission electron micrographs indicate normal sarcomeric assembly (brackets) in juvenile mutants + compared to wildtype controls, however mitochondria (arrows) are noticeably malformed and less abundant. Scale bar 1 µm. (**D**) High magnification electron micrographs showing detailed images of the sarcomeres (brackets) and mitochondria (arrows) in wildtype compared to mutant adolescent muscle samples. Scale bar 200 nm. (**E**) Quantification of mitochondrial area adjacent to sarcomeres showed a statistically significant reduction in mutants (*t-*test) (n = 22 and 17 biological replicates for wild-type and *ppp2r3b−/−* animals, respectively).

Given the link between progressive spinal curvature and proprioception, and the expression of *PPP2R3B/ppp2r3b* that we detected in muscle and bone, we monitored muscle fibre and neuromuscular junction formation in our mutant fish. Initially we analysed skeletal muscle birefringence at 48hpf, an established indicator of muscle fibre integrity based on polarised light transmission through striated fibres. No obvious changes in birefringence signal were detected between *ppp2r3b* mutants and wildtype siblings (Fig. 11A). To challenge a role for PR70 in neuromuscular development, embryos were further stained for F-Actin and Acetycholine receptor (AChR), using fluorescently conjugated phalloidin and α-bungarotoxin. Muscle fibres and AChR localisation appeared organised and indistinguishable between *ppp2r3b*^−/−^ embryos and wild-type siblings (Fig. 11B). Thus, PR70 is not required for normal neuromuscular development.

To evaluate muscle development during juvenile growth, at a point when scoliosis has manifested, transmission electron micrographs were produced from muscle biopsies. *ppp2r3b*^−/−^ fish displayed normal myofibril organisation and sarcomeric assembly (Fig. 11C and D), and consistent with this, qRT-PCR analyses showed that expression of key muscle markers was unchanged (Table 1). However, a marked reduction in mitochondrial content was observed in *ppp2r3b* mutants (Fig. 11C). Closer examination of the mitochondria showed general dysmorphic character in the mutants with undefined cristae and smaller overall size (Fig. 11D). Quantification of mitochondrial area adjacent to sarcomeres showing a statistically significant reduction in mutants (Fig. 11E). Taken together, these data suggest that *ppp2r3b*, whilst not required for the formation of muscle, is required to maintain muscle mitochondrial abundance.

## Discussion

In this study, we have created a targeted mutation in *ppp2r3b* in zebrafish which resulted in juvenile onset, progressive and fully penetrant kyphoscoliosis closely mirroring IS, as well as abnormal mitochondria in the vicinity of axial musculature. Collectively the following pieces of information indicate that this results from a loss of PR70 protein function. While we did not see any differences in transcript levels in mutants by qRT-PCR and no specific antibody is available to detect PR70 protein, we did show that all detectable isoforms of *ppp2r3b* included exon 2, which carries the engineered frameshift mutation. This is located at residue 31 of the encoded protein which would therefore lead to deletion of two EF-hand motifs located within the C-terminal half of the protein which are essential for calcium binding and protein function^6^. Furthermore, protein sequence analysis does not indicate that there are any translation initiation codons downstream of the mutation which could produce protein product not involving the frameshift mutation. Another consideration relates to potential off-target mutations produced by the gene-editing process. Having bred the mutation out for a number of generations, any putative off-target mutations would be lost and certainly not be expected to segregate with the observed phenotype. By contrast, kyphoscoliosis segregated perfectly (100% penetrance) with the frameshift mutation over hundreds of animals and several generations, indicating linkage to the mutation.

The zebrafish has emerged as a powerful model to study IS^4^. By generating a zebrafish mutant in *ppp2r3b*, we have been able to investigate the requirement for this gene beyond larval stages, thereby revealing a requirement for normal bone mineralisation. Two key findings in relation to the potential cellular mechanism of pathogenesis were: (1) the identification of prominent *ppp2r3b* gene expression in somites and axial muscle fibres in contrast to very limited expression within the vicinity of bone, and; (2) the identification of reduced vertebral bone density and altered intervertebral structure, as well as mitochondrial defects similar to those previously reported in muscular dystrophy models which followed normal muscle formation and occurred in the absence of muscle fibre (Z-disc) defects^10^. Both the skeletal and muscle defects that we report were degenerative in nature. Collectively, these observations suggest that kyphoscoliosis occurs secondarily either to defects in muscle function and/or because of disrupted communication between muscle and bone. The holes in the vertebrae, representing sites of low mineralisation were particularly striking. Whether this is a primary cause of IS or an unrelated secondary consequence of loss of spinal integrity remains to be tested. Nonetheless, resembling osteoporosis, this is likely to contribute to the overall pathology. Bending of the spine seems to correlate with intervertebral disc wedging which could be a driving force in pathogenesis, and so the role of PR70 in intervertebral disc formation and maintenance must be investigated further. Expression of PPP2R3B appeared to be mutually exclusive with that of SOX9, a marker of cartilage lineage, at CS-23 but not at CS-22. It is possible that this represents transient co-localization with chondrogenic niches during development and further studies will be required to test this. If so, this may imply that role of ppp2r3b is primarily within the intervertebral discs rather than vertebral bone and will require future investigations. Results from our electron microscopy studies suggest a defect in mitochondria adjacent to muscle fibres, which themselves appeared structurally normal. In future it will be necessary to dissect the causal relatioships between muscle function, mitochondria and vertebral bone/intervertebral disc integrity.

The expression pattern of PPP2R3B in human foetuses is very broad and, at a high level, it looks to be specifically enriched in the nervous system (especially along ventricular surfaces). A growing body of evidence from zebrafish scoliosis models has implicated ependymal cell cilia, circumventricular organ system and cerebrospinal flow defects in the pathogenesis of idiopathic scoliosis. *ppp2r3b* mutant zebrafish embryos do not display features associated with other models of ciliary dysfunction, such as body curvature or gastrulation defects, and we did not detect neuronal defects in our own analyses. Nevertheless, in future mechanistic studies, it will be important to undertake more detailed analyses of neurophysiological origins of scoliosis.

At the molecular level, *PPP2R3B* encodes the DNA origin of replication complex (ORC) regulator, PR70^5,6,8^. Core ORC components, such as CDC6, are mutated in Meier-Gorlin syndrome which features a variety of skeletal defects including scoliosis^11,12^. However, to our knowledge, nothing is known about the molecular or cellular function of the ORC in skeletogenesis. A zebrafish *cdc6* mutant has been reported, but post-larval phenotypes are potentially confounded by severe early embryonic defects and no skeletal features were reported^13^. There was a recently identified role in autophagy as well^7^. The mouse homologue *Ppp2r3a* (mgi 2442104) is reported to have decreased bone mineral content. The zebrafish model that we report here provides a basis to investigate the molecular and cellular mechanisms of pathogenesis in future.

## Materials and methods

### Zebrafish

Zebrafish (Danio rerio) embryos were obtained from a wild-type strain and raised in E3 Medium until the desired developmental stage is reached. This solution contains 5 mM NaCl, 0.17 mM KCl, 0.33 mM CaCl_2_, 0.33 mM MgSO_4_ and 0.1% Methylene Blue. This medium serves as a control medium and will not have any effect on patterning.

### Whole-Mount in situ hybridisation on zebrafish

Chorions were removed manually prior to fixation in 4% paraformaldehyde (PFA) in 1 × PBS overnight at 4 °C. Fixed embryos were dehydrated in 100% Methanol (MeOH) for 15 min. at room temperature. Embryos were cooled to – 20 °C for at least 30 min for permeabilization prior to proceeding with in situ hybridization experiments. Whole-mount in situ hybridization was performed following the protocol from ZFIN. Westerfield^14^.

### In situ hybridisation on human tissue

7 µm paraffin sections were obtained from the Human Developmental Biology Resource (http://www.hdbr.org/). Riboprobes were synthesized with Digoxigenin-UTP RNA labelling kits (Roche) from amplified fragments of each gene using the following primers:

*GFP* (553 bp): EGFP_F, CGACGTAAACGGCCACAAG; EGFP_R, CTGGGTGCTCAGGTAGTGG, using pEGFP-N1 plasmid as template. *PPP2R3B* (556 bp): PPP2R3B_F, CTTCTACGAGGAGCAGTGCC; PPP2R3B_R, TTTACACGAGCCGCGGTG. *SOX10* (561 bp): SOX10_F, AGCCCAGGTGAAGACAGAGA; SOX10_R: TCTGTCCAGCCTGTTCTCCT. Template for PCR amplification of *PPP2R3B* and *SOX10* was human cDNA. The *SOX9* probe has been reported previously^15^. Human embryonic samples were fixed in 10% (w/v) neutral-buffered formalin solution (Sigma-Aldrich) and embedded in paraffin wax before sectioning. ISH was performed in 300 mM NaCl, 5 mM EDTA, 20 mM Tris–HCl, 0.5 mg/mL yeast tRNA, 10% dextran sulfate, 1 × Denhardt’s solution, and 50% formamide with digoxigenin-incorporated riboprobes at 68 °C. Posthybridization slides were incubated with anti-digoxigenin conjugated with alkaline phosphatase (Roche) diluted 1:1000 in 2% newborn calf serum. Expression patterns were visualized with a Nitro-Blue Tetrazolium Chloride/5-Bromo-4-Chloro-3-Indolyphosphate p-Toluidine Salt (NBT/BCIP) system (Roche). Sections were mounted with Vectamount (Vector laboratories) and analyzed with a Zeiss Axioplan 2 imaging system.

### Zebrafish CRISPR/Cas9 mutagenesis using Cas9 plasmid

To create indels in the zebrafish *ppp2r3b* gene, the following sgRNA sequence was selected for targeting (5′ GGAATGCTTTCACTTAAGGCTGG 3′- PAM sequence is underlined). To create the sgRNA, the following 5′ phosphorylated oligonucleotides (5′ **TAGG**AATGCTTTCACTTAAGGC 3′ and 5′ **AAAC**GCCTTAAGTGAAAGCATT 3’) were denatured at 95 °C and annealed by cooling to 25 °C using a 5 °C/min ramp. Oligos were subsequently cloned into pDR274. To generate sgRNAs, the plasmid was linearsied using *Dra I* and transcribed using the Megashortscript T7 kit (Invitrogen, AM1354). Capped RNA encoding Cas9 was synthesised from *XbaI-*linearised pT3TS-nCas9n plasmid using the T3 mMessage mMachine Kit (Ambion). The Poly(A) Tailing Kit (Ambion, AM1350) was used for polyA tailing of RNA. Both sgRNA and Cas9 RNA were purified using the RNeasy mini kit (Qiagen, 74104), which were subsequently co-injected (25 ng/μl sgRNA, 200 ng/μl Cas9 RNA) into zebrafish embryos at the 1 cell stage. To confirm successful mutagenesis total genomic DNA was extracted from individual 24 hpf embryos in 50 mM NaOH at 95 °C for > 10 min, which was subsequently neutralised in 1 M Tris–HCl, pH 8.0. PCR was performed using the Phire Animal Tissue Direct PCR kit (Thermo Scientific F-140WH), which was subject to direct sequencing, T7 Endonuclease I assay (T7EI; NEB M0302L) or restriction enzyme digestion using *Mse I* (NEB R0525L). Splicing analysis of exon 2 was done using primers in exons 1 and 3 or exons 1 and 7, generating RT-PCR prodcuts of sizes 491 bp and 1000 bp, respectively. Primer sequences were as follows: ZFcDNA ppp2r3b Exon1_F aagggcacaagcactttgat, ZFcDNA ppp2r3b Exon3_R tttctccacattgccacaaa, cDNA_ppp2r3b Exon_1_F cttttggttgagtgagccgag and cDNA_ppp2r3b Exon_7_R atgccgcattaggtctctctg.

### Zebrafish CRISPR/Cas9 mutagenesis using RNPs

Three *ppp2r3b* targeting ribonucleotide protein (RNP) complexes were injected into zebrafish embryos to disrupt *ppp2r3b* function, increasing the likelihood of recovering targeted mutations. 2 nmol crRNAs were synthesised against exon1 (IDT, CD.Cas9.SQVS6625.AL: CAAAGATTAATGCAACGGAG), (IDT, CD.Cas9.SQVS6625.AA : AGGACTGCACGGGGCACCAA), and exon 3 (IDT, CD.Cas9.SQVS6625.AI: ATGGAGCTTTCCAATAGAAT). RNP complexes were established following an adapted protocol from Kroll et al.^16^. In brief, 2 nmol crRNA for each target was resuspended in 10 µl duplex buffer (IDT, #11-01-03-01). 5nmole tracrRNA (IDT, #1072532) was resuspended in 25 µl Duplex buffer. Each crRNA was annealed to the tracrRNA by combining 1 µl of crRNA with 1 µl of tracrRNA and 1.5 µl of duplex buffer, components were incubated at 95 °C in a thermocycler. The Cas9 was then complexed to the annealed guide RNAs by adding 1 µl of 10 µg/µl Cas9 endonuclease (Alt-R S.p. HiFi Cas9 nuclease, IDT, #1081060) to each of the 3.5 µl guide RNA solutions and incubated at 95 °C for 5 min. All three complexed RNPs were then pooled, mixed, aliquoted and stored at − 20 °C until required. 0.5 nl of pooled RNPs were intracellularly microinjected into wildtype (AB × TupLF) embryos at the one-cell stage and embryos allowed to develop until adulthood.

### Morphometric analyses

Zebrafish were fixed in 10% neutral buffered formalin (NBF) for 24 h and stored in 70% ethanol until scanning. Alcian blue and Alizarin red staining was performed as described previously^17^. X-ray Micro Computed Tomography (µCT) analysis of cortical bone parameters was performed on the 1st caudal vertebra (SkyScan 1172, Bruker, Belgium). A negative offset of around 10 sections (0.01 mm) was applied in the selection of the vertebral bone from either extremity. The µCT scanner was set at 40 kV and 250 µA, the image was acquired at a camera resolution of 2000 × 1048 using no filter and a pixel size of 1.85 µm (for the high-resolution vertebral analysis). For the whole Zebrafish scans the pixel size was set at 11.31 µm. The images were reconstructed, analysed and 3D rendered using the following software: NRecon, CTAn, CTVox, respectively (Bruker, Belgium). A blind analysis of the following cortical bone parameters was performed: cortical bone volume % (BV/TV), cortical bone thickness (B.Th.), vertebral bone length and diameter, bone mineral density (BMD) and tissue mineral density (TMD). Flat-field corrections were applied to each scan and hydroxyapatite calibration phantoms of 0.25 g/cm^3^ and 0.75 g/cm^3^ were used for reference. For all analyses presented in the manuscript, mutant and wild-type animals were matched to tail length. (Bone histomorphometry nomenclature was based on the expressions used by Parfitt et al. 1987).

Details of SKYSCAN 1172 micro-CT image acquisition setting are as follows:

Source voltage (kV) = 40.

Source current (uA) = 250.

Camera image resolution: 2000 × 1048.

Pixel size (um) = 1.86.

Filter = No filter.

Image format = TIFF.

Depth (bits) = 16.

Screen LUT = 0.

Exposure (ms) = 590.

Rotation step (deg) = 0.500.

Frame averaging = ON (3).

### TEM on zebrafish

Tissue was rinsed in 4 ml cacodylate buffer (0.1 M, pH 7.2–7.4) and fixed in 2.5% glutaraldehyde in 0.1 M Cacodylate buffer pH7.2. Tissue was rinsed twice in 4 ml cacodylate buffer (0.1 M, pH 7.2–7.4) for 15 min and post-fixed in 2% osmium tetroxide (Agar scientific) for 1 h at room temperature. Tissue was rinsed again twice in 4 ml cacodylate buffer (0.1 M, pH 7.2–7.4) for 15 min and dehydrated through a graded series of ethanol (70%, 90%, Absolute), transferred into propylene oxide (3 × 10 min) and into a 1:1 propylene oxide/resin mix (Agar 100, Agar Scientific) for 1 h. Tissue was then put into resin for 1 h on a rotator before being transferred into coffin molds and placed in the oven to polymerise for 48 h. Semi thin sections (0.5 µm) were cut using a Reichert-Jung ultracut E microtome and stained with toluidine blue for 2 min to check tissue orientation. Thin sections (80–100 nm) were cut using a Reichert-Jung ultracut E microtome and collected on copper mesh grids (Agar Scientific). They were then stained with 2% uranyl acetate in aqueous solution (Taab Laboratories) for 15 min followed by lead citrate stain^18^ for 2 min. Samples were examined using a Hitatchi 7100 electron microscope (Wokingham, Berks, UK) operating at 75 kV.

For quantification either area of visible mitochondria or integrated pixel density was used. According to image J *"Integrated density - The sum of the values of the pixels in the image or selection. This is equivalent to the product of Area and Mean Gray Value". *This is informative as it tells us how much EM-signal is captured in a given area, which equates to mitochondrial coherence/integrity. We were interested in the coverage of signal within the mitochondria rather than signal brightness. Mutant mitochondria appeared less dense and were more dissociated in structure, which this tool addresses.

### Analysis of muscle in zebrafish

Skeletal muscle birefringence was used to evaluate muscle fibre integrity.

Muscle birefringence in live embryos was visualized using a Nikon SMZ1270 dissecting microscope with a C-POL polarizing attachment.

Phalloidin-568 (Molecular Probes, A12380) and alpha-bungarotoxin-488 (Invitrogen, B13422F) stain was used to interrogate muscle structure (F-Actin) and neuromuscular junctions (Acetylcholine Receptors), respectively. Embryos were fixed overnight in 4% paraformaldehyde at 4 °C. Washed in PDT (PBS containing 1% DMSO and 0.8 × Triton), proteinase K treated (10 μg/ml) for 30 min, washed again in PDT before being incubated overnight in 1:80 Phalloidin-568 (from a 12uM stock dissolved in methanol) and 1:1000 alpha bugarotoxin-488 (from a 1 mg/ml stock dissolved in PBS). Embryos were further washed in PDT, mounted in CitiFluor AF1 media (Agar Scientific, AGR1320) and imaged using a Nikon A1R confocal microscope in SGUL’s Image Resource Facility.

### Ethics statement

All methods were carried out in accordance with relevant guidelines and regulations. Animal maintenance, husbandry, and procedures are defined and controlled by the Animals (Scientific Procedures) Act 1986. All animal procedures were authorised by Home Office Licences PPL:70/7892 and PPL: P3DFD4131, in compliance with the Biological Services Management Group and approved by the Biological Services Ethical Committee, UCL, London, UK. All metnods are reported in accordance with ARRIVE guidelines. Human embryonic samples were obtained from the Human Developmental Biology Resource (HDBR; http://www.hdbr.org/) with appropriate written informed consent from the donor and ethical approval from Fulham Research Ethics Committee (18/LO/022) and North East-Newcastle & North Tyneside 1 Research Ethics committee (18/NE/0290). The HDBR is regulated by the UK Human Tissue Authority (www.hta.gov.uk) and operates in accordance with the Human Tissue Authority Codes of Practice.

## Supplementary Information

Supplementary Figure 1.

## Data Availability

All data sets and raw data are provided in this manuscript and are available to all readers. Please contact the corresponding author for further information if required.
