# Supplementary material for: A CRISPR/Cas9-generated mutation in the zebrafish orthologue of PPP2R3B causes idiopathic scoliosis
Source: Sci Rep. 2023 Apr 26;13:6783. doi: 10.1038/s41598-023-33589-y (PMC10133272; doi:10.1038/s41598-023-33589-y)
Supplement: Supplementary file 1 — Supplementary Figure 1. [file 41598_2023_33589_MOESM1_ESM.docx]

**Supplementary figures**

**
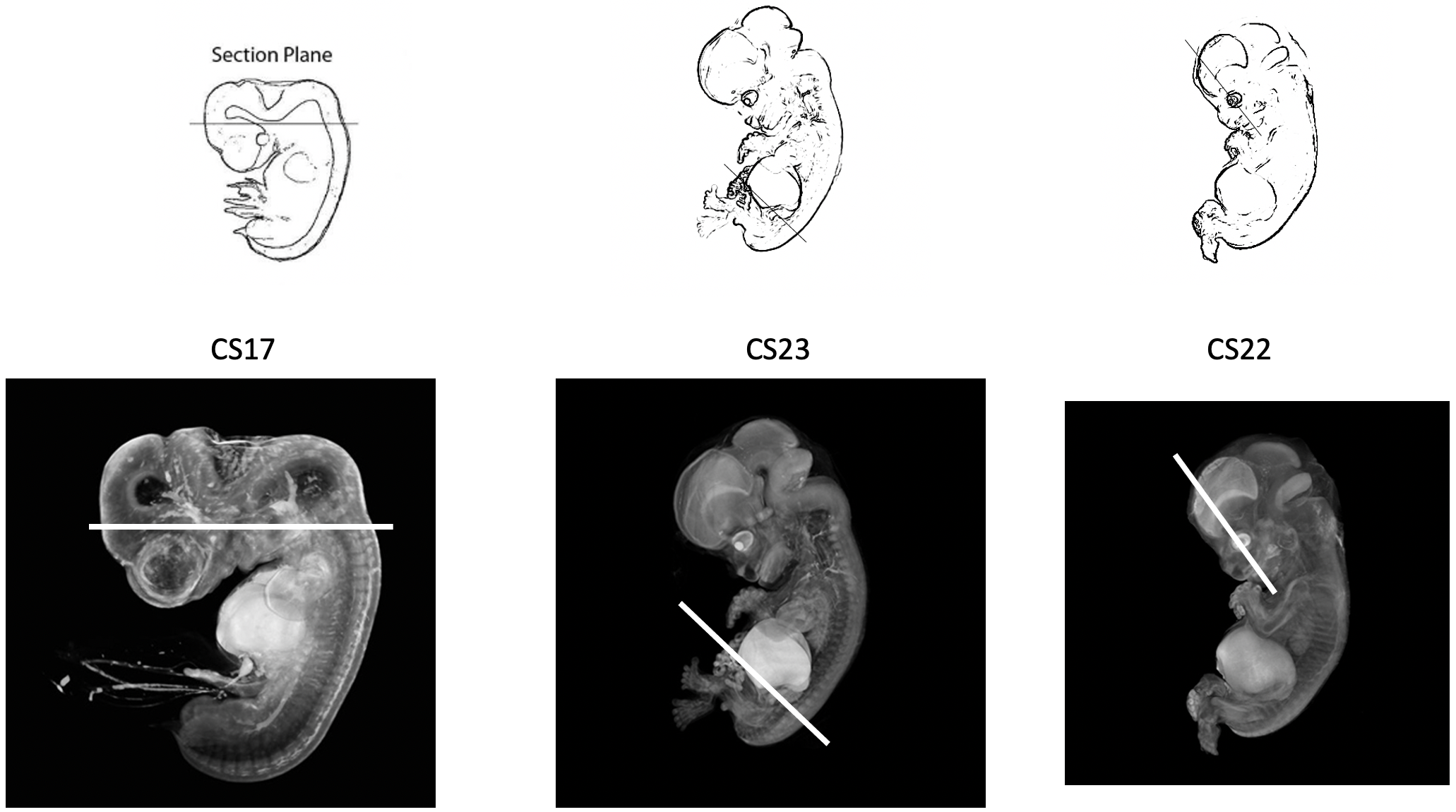
**

**Supplementary Figure 1. Orientation of sections analysed in Figure 1.**

The CS22 and 23 models are micro CT scans. Sample staining and micro-CT were performed by Newcastle [Preclinical In Vivo Imaging (PIVI)](https://eur01.safelinks.protection.outlook.com/?url=https%3A%2F%2Fwww.ncl.ac.uk%2Fpivi%2F&data=05%7C01%7Cm.seda%40ucl.ac.uk%7Cb9aea0958e754889489308db2c5ecb3b%7C1faf88fea9984c5b93c9210a11d9a5c2%7C0%7C0%7C638152557870992769%7CUnknown%7CTWFpbGZsb3d8eyJWIjoiMC4wLjAwMDAiLCJQIjoiV2luMzIiLCJBTiI6Ik1haWwiLCJXVCI6Mn0%3D%7C3000%7C%7C%7C&sdata=iqmE5QhDHk9XE2yXWJ91Ho7%2FgUjRlaOQhWnjlMzb3Kw%3D&reserved=0)  facility. 3D data-sets were acquired using Skyscan 1176, Bruker-microCT. The CS17 is an Optical Projection Tomography model <https://www.science.org/doi/10.1126/science.1068206>

Images reproduced with permission of the HDBR atlas. [https://hdbratlas.org/carnegie_stages.html](https://eur01.safelinks.protection.outlook.com/?url=https%3A%2F%2Fhdbratlas.org%2Fcarnegie_stages.html&data=05%7C01%7Cm.seda%40ucl.ac.uk%7Cb9aea0958e754889489308db2c5ecb3b%7C1faf88fea9984c5b93c9210a11d9a5c2%7C0%7C0%7C638152557870992769%7CUnknown%7CTWFpbGZsb3d8eyJWIjoiMC4wLjAwMDAiLCJQIjoiV2luMzIiLCJBTiI6Ik1haWwiLCJXVCI6Mn0%3D%7C3000%7C%7C%7C&sdata=jxXyTqxNL79HpVxfrS%2Bfjejn8OPibHi9tdK3DEFUoWE%3D&reserved=0))

Drawings were provided by Janet Kerwin.
